# Supplementary material for: A Functional Analysis of the Unclassified Pro2767Ser BRCA2 Variant Reveals Its Potential Pathogenicity that Acts by Hampering DNA Binding and Homology-Mediated DNA Repair
Source: Cancers (Basel). 2019 Sep 28;11(10):1454. doi: 10.3390/cancers11101454 (PMC6826418; doi:10.3390/cancers11101454)
Supplement: Supplementary file 1 [file cancers-11-01454-s001.pdf]

Supplementary Materials: A Functional Analysis of the Unclassified Pro2767Ser *BRCA2* Variant Reveals its Potential Pathogenicity that Acts by Hampering DNA Binding and Homology-Mediated DNA Repair

Maria Valeria Esposito, Giuseppina Minopoli, Luciana Esposito, Valeria D'Argenio, Federica Di Maggio, Emanuele Sasso, Massimiliano D'Aiuto, Nicola Zambrano and Francesco Salvatore

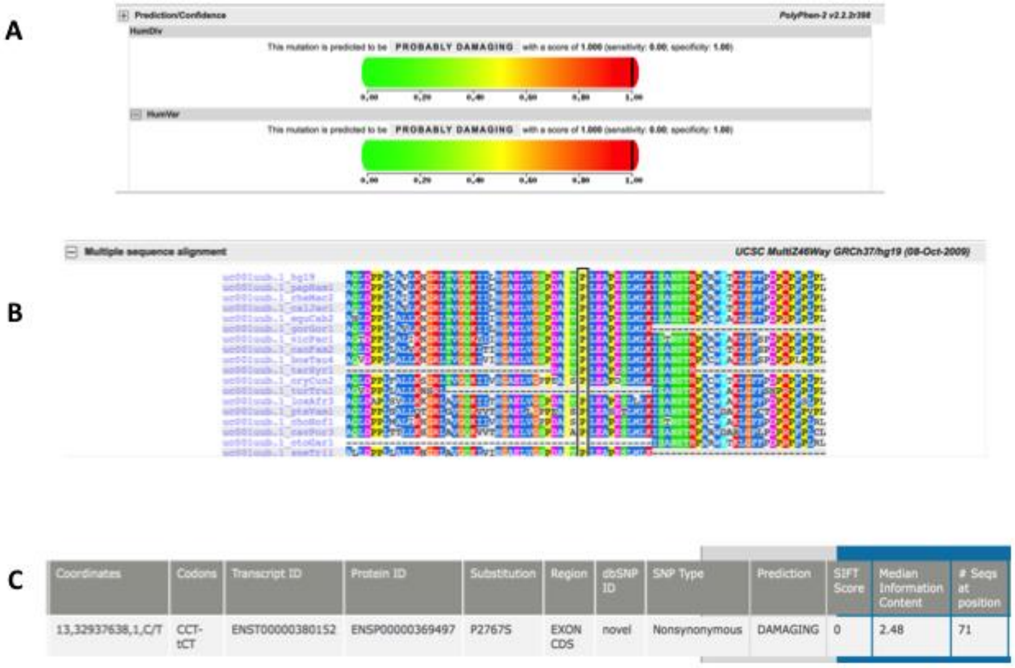

Coordinates	Codons	Transcript ID	Protein ID	Substitution	Region	dbSNP ID	SNP Type	Prediction	SIFT Score	Median Information Content	# Seqs at position
13,32937638,1,C/T	CCT-tCT	ENST00000380152	ENSP00000369497	P2767S	EXON CDS	novel	Nonsynonymous	DAMAGING	0	2.48	71

Figure S1. In silico pathogenicity prediction and multiple sequence alignment variant derived from PolyPhen-2 and SIFT software. (**A**) PolyPhen-2 prediction scores. (**B**) Alignments of the amino acid residue affected by the variant with evidence of high conservation between species. (**C**) SIFT prediction of variant pathogenicity.

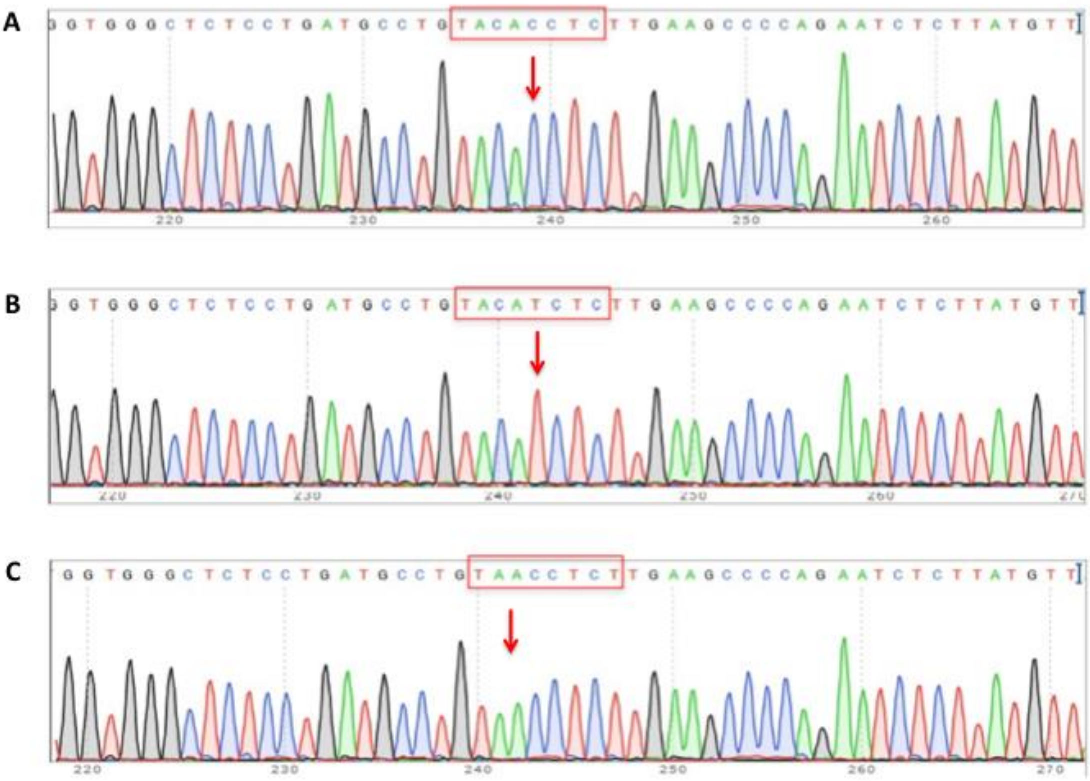

Figure S2. Mutagenesis reactions results evaluated by standard Sanger sequencing. (**A**) Electropherogram showing the wild-type nucleotide C at position 8299 of BRCA2 cDNA. (**B**) Substitution C > T to reproduce the Pro2767Ser variant at c.8299. (**C**) Deletion del C at c.8297 nucleotide position to obtain Thr2766Asnfs.

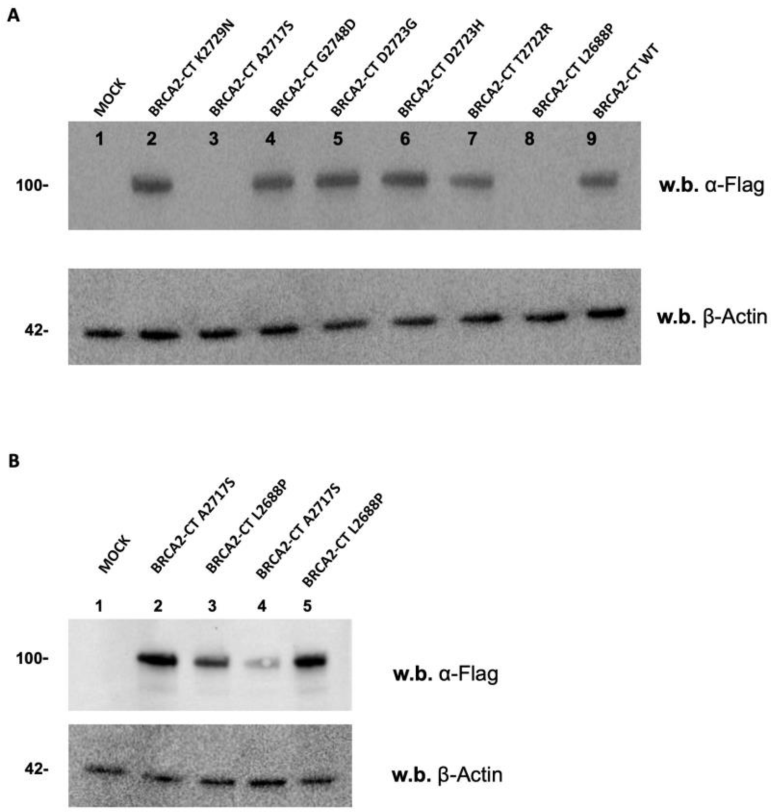

Figure S3. Western Blot analysis of all BRCA2-CT minigenes for validation set analysis. (**A**) All BRCA2-CT CTRs showed proper expression in NIH-3T3 cells. (**B**) Further transfections to assess the expression of the remaining BRCA2-CT A2717S and L2688P controls. A mutant BRCA2 protein of 95kDa was detected in all samples.

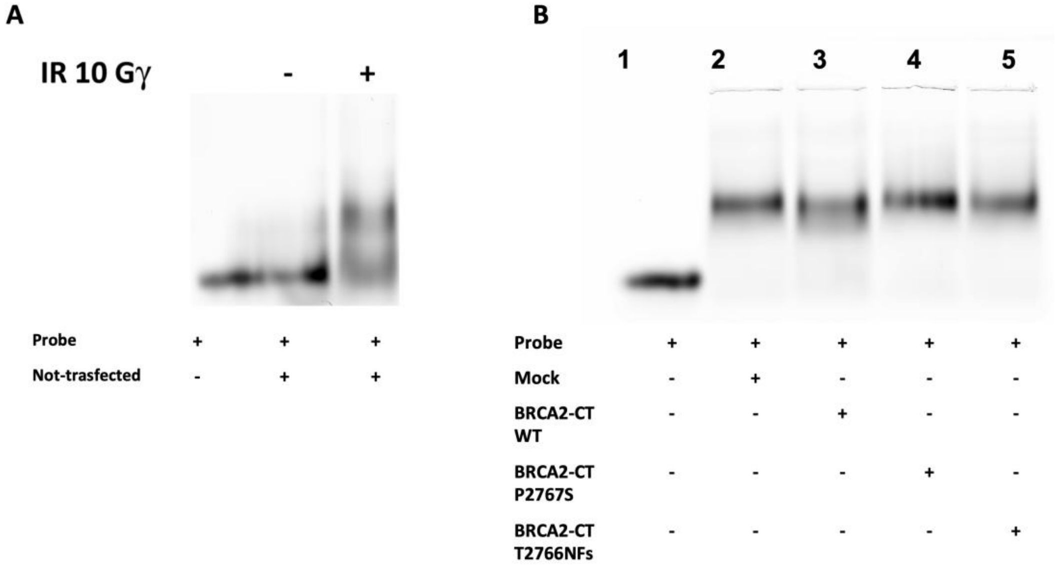

Figure S4. (**A**) In vitro ssDNA binding assay of not-transfected not-treated (NT) and not-transfected irradiated (IR) cells. Migration was not delayed in NT nuclear extracts and in the free probe but there was a slow migrating band in IR nuclear extracts, thereby demonstrating activation of the ssDNA-binding system. (**B**) In vitro ssDNA binding assay of BRCA2-CT WT, P2767S and T2766NFs without IR. All samples show similar band-migration delay, including BRCA2-CT WT-transfected cells that behaved differently respect to BRCA2-CT WT IR sample. This suggests that the damage-sensitive proteins were activated also by transfection procedures, such as by the serum stimulation or by cell replication mechanisms.

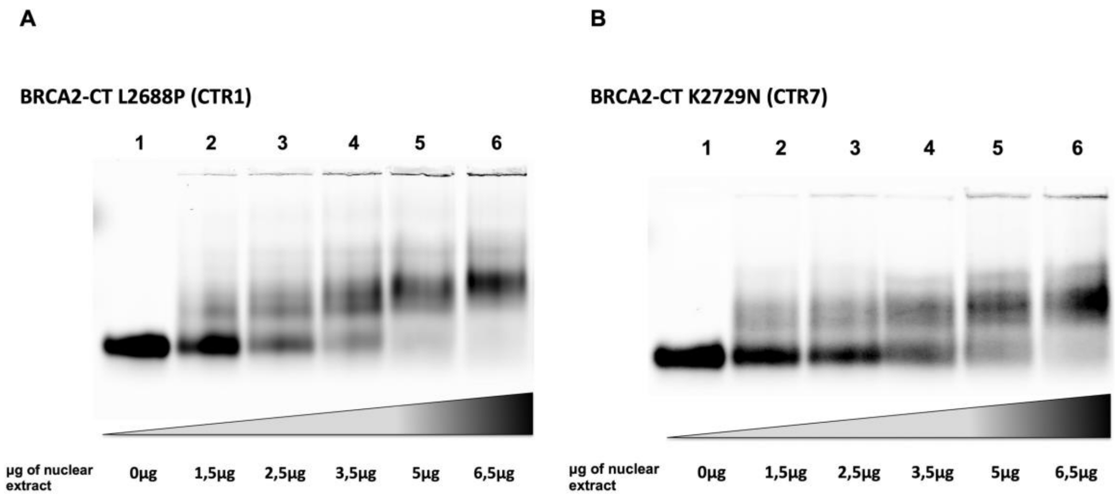

Figure S5. Dose dependent binding assay of the BRCA2-CT L2688P (**A**) and of the BRCA2-CT K2729N (**B**) samples.The ssDNA-binding assay was performed using increasing amount of the nuclear extract. For each sample was loaded: the binding reaction with only free probe, 1.5 µg, 2.5 µg, 3.5 µg, 5 µg, and 6.5 µg of nuclear extract of irradiated cells. The DNA-protein complex formation increases when more protein is present. In particular, differences between BRCA2-CT L2688P and BRCA2-CT K2729N are clearly shown from 5 up to 6.5 µg of nuclear extract (lanes 5–6).

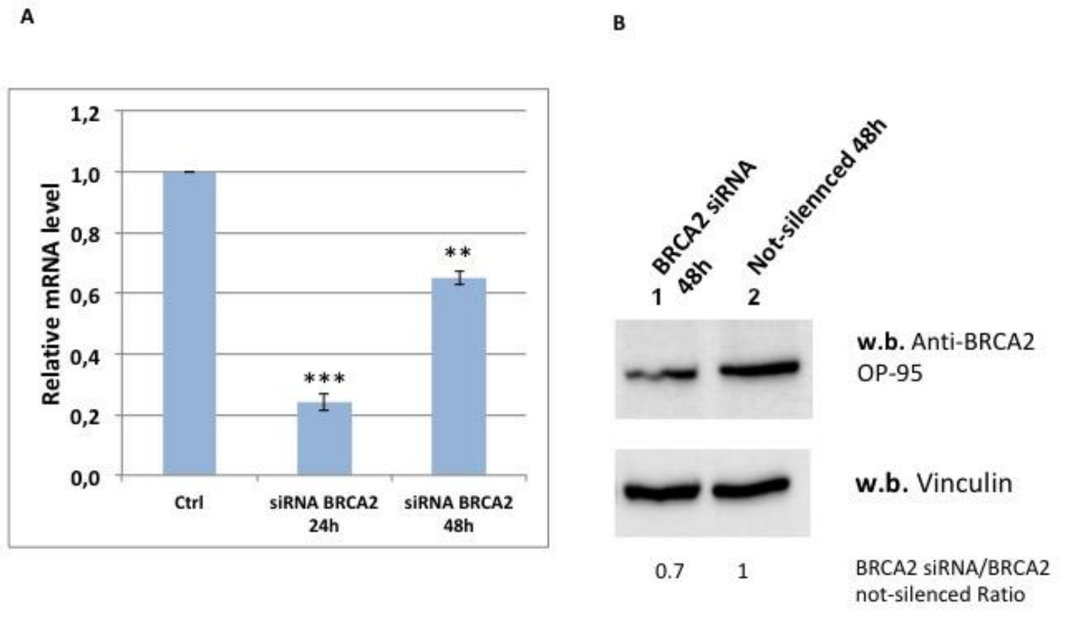

Figure S6. The RNAi results were evaluated by quantitative real-time PCR (qPCR) and Western Blot analysis. (**A**) Real-time qPCR analysis. The mRNA levels of BRCA2 were determined by real-time qPCR and normalized by the Actin mRNA level. Data are expressed as relative to the values obtained on transfection with siRNA negative control. Bars represent means ± sd. p-values denoted are reported as follows: ns ($p > 0.05$), * ($p \le 0.05$), ** ($p \le 0.01$), *** ($p \le 0.001$), **** ($p \le 0.0001$). (**B**) Western Blot Analysis of BRCA2 endogenous protein. The ratio of silenced BRCA2 to not-silenced signal is reported at the bottom.

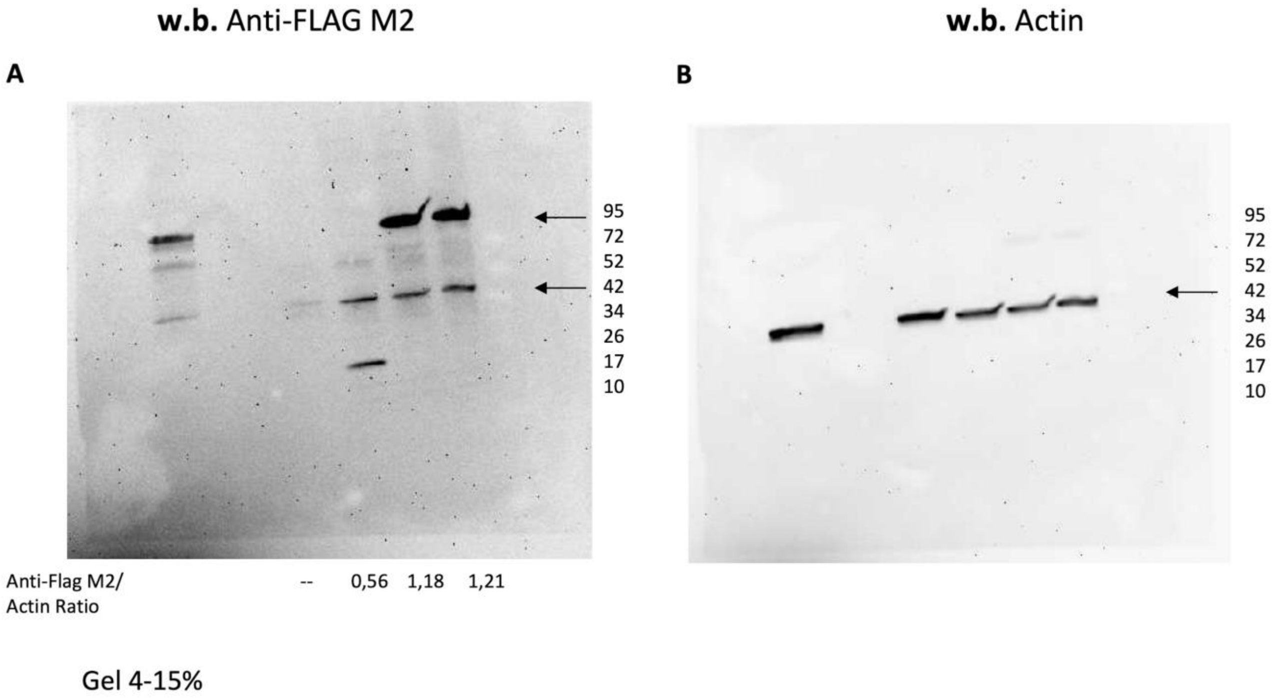

Figure S7. Figure 3C: Western blot analysis of the expression of BRCA2-CT wt, BRCA2-CT P2767S and BRCA2-CT T2766NFs proteins in NIH-3T3 cells. A mutant BRCA2 protein of 95kDa was detected in cells that contained BRCA2-CT wt and BRCA2-CT P2767S proteins; a 15kDa deleted BRCA2-CT T2766NFs protein derived from the Thr2766AsnFs mutation. On the left (A) the entire gel in which are shown the BRCA2-CT flagged bands detected by Anti-Flag M2 (mouse, 1:1000) antibody. On the right (B) the Actin was used as loading control (mouse, 1:1000). The ratio of BRCA2-Flag to the Actin signal is reported at the bottom. The gel was a 4-15% precast. Bands were detected by exposure to the ChemiDoc MP System (Bio-Rad).

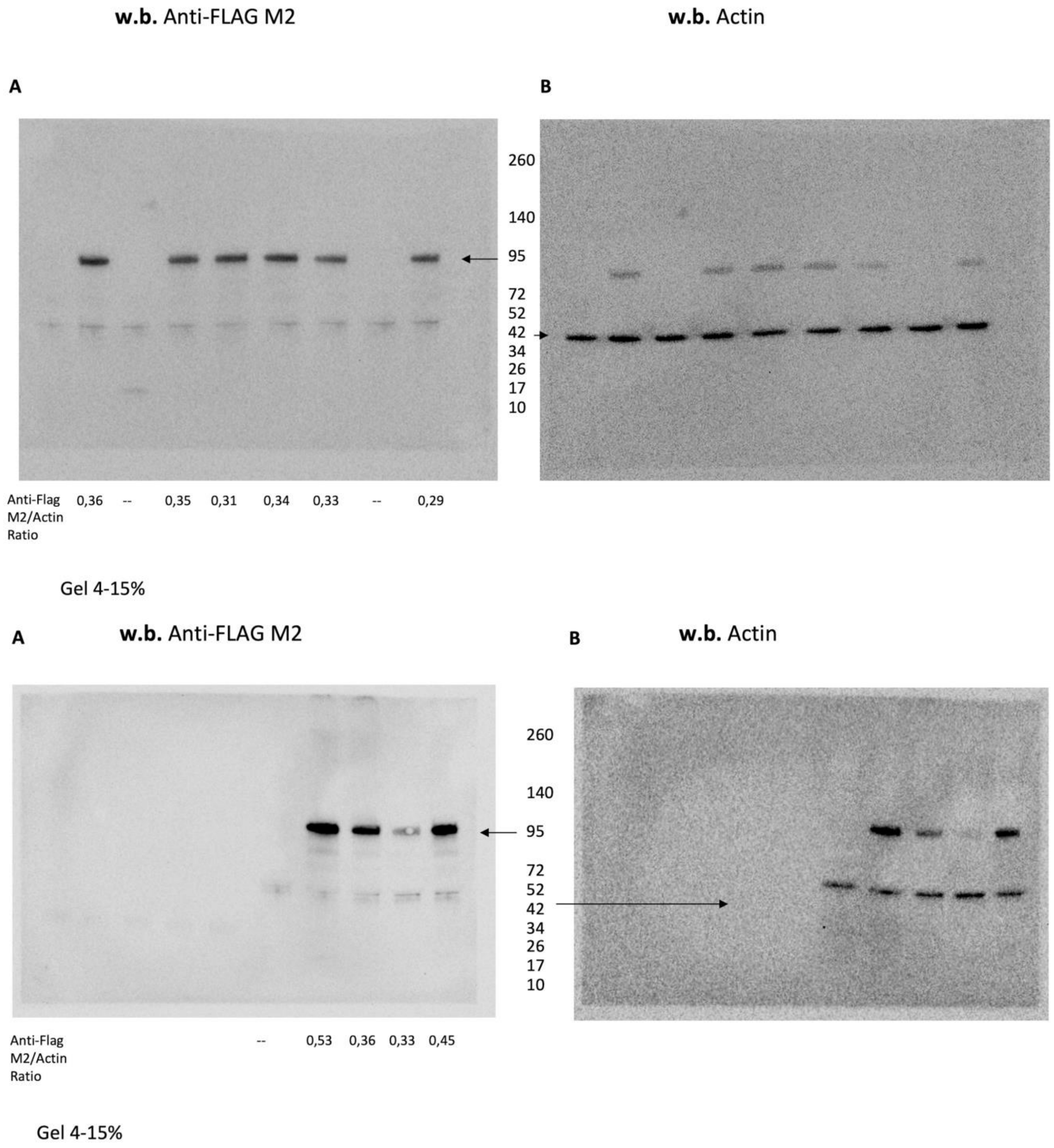

Figure S8. Figure S3 A and B: Western Blot analysis of all BRCA2-CT minigenes for validation set analysis. All BRCA2-CT CTRs showed proper expression in NIH-3T3 cells. A mutant BRCA2 protein of 95kDa was detected in all samples. Respectively, on the left the entire gels in which are shown the BRCA2-CT 95kDa flagged bands detected by Anti-Flag M2 (mouse, 1:1000) antibody. On the right the Actin was used as loading control (mouse, 1:1000). The ratio of BRCA2-Flag to the Actin signal is reported at the bottom. The gel was a 4-15% precast. Bands were detected by exposure to the ChemiDoc MP System (Bio-Rad).

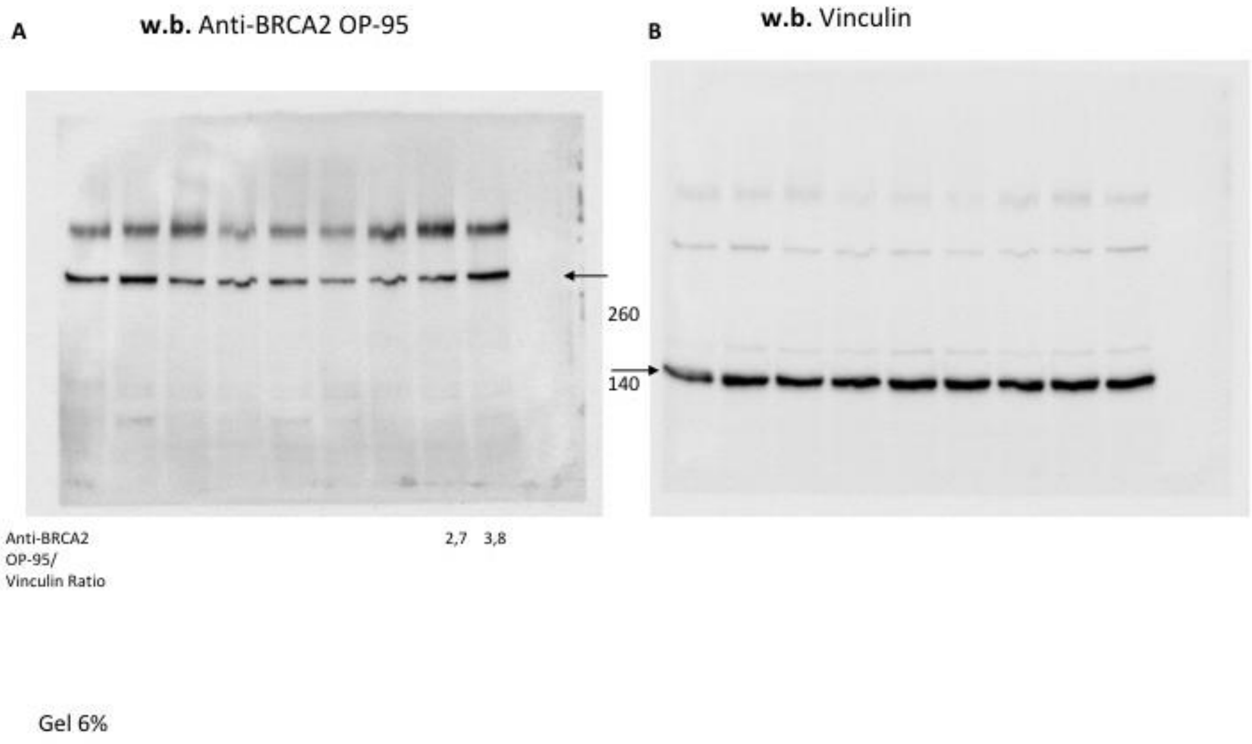

Figure S9. Figure S6 B: The RNAi results evaluated by Western Blot analysis 48 hours after transfection. On the left (A) the entire gel in which are shown the endogenous BRCA2 bands detected by Anti-BRCA2 OP-95 (Ab-1) Mouse mAb 2 (1:1000) antibody. On the right the Vinculin of about 130 kDa (goat, 1:1000) was used as loading control. The ratio of BRCA2 to the Vinculin signal is reported at the bottom. The gel was a 6% polyacrylamide gel. Bands were detected by exposure to the ChemiDoc MP System (Bio-Rad).

Table S1. Sequence of primers used for cloning and in vitro ssDNA binding reactions.

	Primer Names	Primer Sequences
a	**BRCA2_DBD _Fw_Not**	5′-AGTTCAGCGGCCGCAGTCAGAATGGAATGTGCCTTTCCTAAGGAATTTGCTA-3′
b	**BRCA2_DBD _Rev_BamHI**	5′-AGAggatccTTAGATATATTTTTTAGTTGTAATTGTGTCCTGC-3′
c	**Linear φX174 ss-DNA**	5′-GGGCGAATTGGGCCCGACGTCGCATGCTCCTCTAGACTCGAGGAATTCGGTACCCCGGGTTCGAAATCGATAAGCTTACAGTCTCCATTTAAAGGACAAG-3′

Table S2. Sequence of primers used for qPCR analysis.

	Primer Names	Primer Sequences
a	**BRCA2_mouse_Fw**	5′-CGAGATGCAGCACAGCAGATTTAGGACCG-3′
b	**BRCA2_mouse_Rev_**	5′-CCACAGCTGTTTAAAACACCACAGAGG-3′
c	**Actin_mouse _Fw_**	5′-CTAAGGCCAACCGTGAAAAGAT-3′
d	**Actin_mouse_Rev**	5′-′GCCTGGATGGCTACGTACATG-3′

© 2019 by the authors. Licensee MDPI, Basel, Switzerland. This article is an open access article distributed under the terms and conditions of the Creative Commons Attribution (CC BY) license (http://creativecommons.org/licenses/by/4.0/).
